# Adding the neuro to cognition: from food storing to nest building

**DOI:** 10.1007/s10071-022-01725-2

**Published:** 2022-12-08

**Authors:** Susan D. Healy

**Affiliations:** Centre for Biological Diversity, School of Biology, University of St Andrews, St Andrews, KY16 9TH UK

**Keywords:** Brain size, Food storing, Hippocampus, Navigation, Nest building, Spatial learning and memory

## Abstract

Typically, investigations of animal cognition couple careful experimental manipulations with examination of the animal’s behavioural responses. Sometimes those questions have included attempts to describe the neural underpinnings of the behavioural outputs. Over the past 25 years, behaviours that involve spatial learning and memory (such as navigation and food storing) has been one context in which such dual or correlated investigations have been both accessible and productive. Here I review some of that work and where it has led. Because of the wealth of data and insights gained from that work and song learning before it, it seems that it might also be useful to try to add some neurobiology to other systems in animal cognition. I finish then, with a description of recent work on the cognition and neurobiology of avian nest building. It is still relatively early days but asking questions about the cognition of nest building has already shown both neural correlates of nest building and that learning and memory play a much greater role in this behaviour than previously considered. While it is not yet clear how putting these components together will be synergistic, the examples of song learning and food storing provide encouragement. Perhaps this might be true for other behaviours too?

## Introduction

The past quarter decade has witnessed a flowering of animal cognition, with an ever-increasing diversity of species tested and with ever more experiments being conducted on animals in the wild (Healy 2019). Identifying and quantifying roles for cognition in a widening variety of behavioural contexts is also becoming commonplace, and the fields of animal cognition and behavioural ecology frequently intersect. In particular, there are increasing examples in which studies begin with a functional question that leads to predictions about the mechanistic basis of the animals’ behaviour. Just three examples of the proverbial tip of this cognitive iceberg are: Muth et al. (2021) tested whether the different foraging roles played by female and male bumblebees *Bombus* lead to sex differences in associative learning in these bees (no, apparently not), Kjernsmo et al. (2019) examined the relative effectiveness of the size and contrast of eyespots of prey on reducing predation rates by three-spined sticklebacks *Gasterosteus aculeatus* (eyespot size seems more effective than eyespot contrast), while Clement et al. (2017) suggest that guppies *Poecilia reticulata* from high predation sites pay relatively more attention to predators than to food in comparison with guppies from low predation sites.

What has been, and continues to be, less evident, however, as animal cognition has matured over the past 25 years is much of an enthusiasm to couple behavioural measures of cognition together with other mechanisms, specifically the neural bases of cognitive performance. There are, however, conspicuous exceptions, among them the use of brain size as a proxy for cognition and investigations of the relationship between spatial learning and memory and hippocampal structure and function.

## Brain size as a proxy for cognition

Lefebvre et al. (1997) drew attention to the potential of brain size data to enable ‘operational’ access to the cognitive abilities of a wide range of species, and species not typically kept in captivity. Many have since found their argument compelling and for a long while brain size has stood in for, or has been used as confirmation of, explicit testing of cognitive abilities (e.g. Horschler et al. 2019; MacLean et al. 2012; Tait et al. 2021). If brain size *is* a good/useful proxy for cognition, it could allow access to cognitive assessments of a much greater diversity of species, perhaps even to those no longer with us (e.g. dinosaurs: Knoll et al. 2021; baleen whales: McCurry et al. 2021; primates: van Schaik et al. 2021).

As I have argued elsewhere (e.g. Healy 2021), before one can readily take brain size as a useful proxy for cognition one might wish to consider whether brain size data fit the bill well enough i.e. what does brain size mean? One might ask what ‘stuff’ makes up a brain, and more importantly for animal cognition researchers how that ‘stuff’ results in the cognitive performance we see in our animals. For example, modularity of neural function shows that not all parts of the brain are involved in what is typically accepted as ‘cognition’ albeit those regions may have connections to parts of the brain that are (e.g. Pessoa 2010). For example, regions such as the amygdala, the hypothalamus, the motor cortex, are respectively involved in emotion, hormonal control, and control of motor output such as coordination and dexterity, rather than in cognition directly. Additionally, while technological advances allow increasingly better visualisation of brain structure such as the structural magnetic resonance imaging (MRI) data from 33 dog breeds (Hecht et al. 2019), the function of some apparently ‘cognitive’ regions remain under-described and their role in cognitive performance is almost entirely correlative (e.g. the avian nidopallium, Bloomston et al. 2022; Rook et al. 2021; the vertebrate cerebellum, Sokolov et al. 2017). Using the size of the whole brain, then, is at best as very crude measure of cognition, and one that would require cognition to be defined in its very broadest sense of information processing (e.g. beyond Shettleworth 2010).

Neural modularity also calls into question the ways in which brain size is typically measured. It might seem antediluvian, but quite a lot of the available whole brain data continues to come, not from brains themselves, but from filling skulls with lead shot or similar (e.g. Isler et al. 2008). Such measures have to ignore any notion of modularity, or assume it is relatively unimportant. Even methods that are much more *du jour* such as the isotropic fractionator method of neuron counting (e.g. Herculano-Houzel et al. 2015), when applied to whole brains also cannot deal with modularity. The more recent applications of cell counting, which are now being done at the level of specific brain regions with known function, seem much more likely to enable coupling neural quantification together with behavioural performance (e.g. Jacob et al. 2021; Strockens et al. 2022).

It seems ironic that as we get closer to measures of brain size becoming useful proxies for cognitive abilities, the need for brain size to substitute as a measure for such cognitive abilities seems to be rapidly reducing. As noted above, the 25 years since Lefebvre et al.’s paper has seen a marked increase in the diversity of species now accessible to cognitive testing, including multiple species in the wild (e.g. alpacas: Abramson et al. 2018; urban raptors: Biondi et al. 2022; lions: Borrego 2020; a threatened gull species: Castano et al. 2022; Asian elephants: Jacobson et al. 2022; bumblebees: Mirwan and Kevan 2014; wild African striped mice: Rochais et al. 2022; fawn-footed mosaic-tailed rats: Rowell and Rymer 2022; dingoes: Smith and Litchfield 2010; brush-tailed possums: Wat et al. 2020). Also importantly, although these tests are still mostly directed at innovation and problem solving as recommended by Lefebvre et al., they are increasingly being addressed to tests that are more explicitly tests of cognitive performance (e.g. Tebbich et al. 2016; van Horik et al. 2020). In this increasing mapping of cognitive abilities, however, there is a noticeable deficit in the amount of attention directed towards investigating problem solving or cognition in invertebrate species (Collado et al. 2021; Eckert et al. 2022; Perry and Chittka 2019; Pfeffer and Wolf 2020; Philips et al. 2017).

## Neuroanatomical correlates of cognitive performance

If there has been so much progress in both accessing cognitive performance in more species, and in increasing the precision of quantifying the neural bases of that performance, perhaps this is all the neuro that might be added to cognition? For those especially interested in cognitive abilities rather than in their neural underpinnings, there are plenty of questions that can be addressed very usefully without poking around in the brains of their subjects. Here, however, I would like to argue that our understanding of cognitive abilities, both functionally and mechanistically, can benefit from such ‘poking around’. Song learning and imprinting are the best exemplars of such benefits. In the context of song, the behavioural data showed us whose songs young birds learn, when they learn, what they learn, how they do or do not modify their songs through their lifetime, and lots more (e.g. Slater et al. 1993, 1991; Slater and Jones 1998). The template model for learning helped to direct experiments that involved looking at the role of auditory feedback (young birds held in isolation or deafened, so they heard either just their own voices, or no voices). Sensitive periods for learning were identified and, as for filial and sexual imprinting, became part of the weft of our understanding of the role of development in behaviour, including into adulthood. The accompanying song-system neural data, on the other hand, have enabled us to see that brain structure, including neuron numbers, changes through early development, as the behaviour of song learning was also moving through the stages of babbling, plastic song, and onto crystallised song, differs between the sexes, and, again lots more (e.g. Balthazart and Ball 1995; Daou and Margoliash 2020). Hand in hand the two approaches to understanding song learning directed which neural and the behavioural questions could and should be addressed, and putting them together has enabled considerable advances not just in understanding song learning but also in, amongst others, the evolution of song and human language (e.g. Beltman et al. 2003; Freeman et al. 2017; Searcy and Nowicki 2019), and the role of sleep in memory consolidation (e.g. Margoliash and Schmidt 2010; Shank and Margoliash 2009).

The contributions made by the work on song learning (and imprinting) are, however, rarely included in the consideration of animal cognition (at least as exemplified by animal cognition textbooks). One example that has more frequently appeared in animal cognition texts is the outcome of the combining together of food storing, spatial memory, and hippocampal structure and function (Olmstead and Kulhlmeier 2015; Shettleworth 2010; Wynne and Udell 2013).

As had happened with song learning, that work began with a behaviour: food storing in a handful of songbirds (particularly tits, chickadees, and corvids, e.g. Bossema 1979; Cowie et al. 1981; Haftorn 1956, 1974; Tomback 1980). Observers of these birds’ foraging saw that when these birds encountered excess food (seeds, nuts) they did not eat them all, but took some away and hid them. Hiding food away as a means to reduce variability in future food access has evolved in only some birds (and other animals), perhaps because for them only storing such food on their bodies as fat is more difficult (Andersson and Krebs 1978; Pravosudov and Grubb 1997; Roberts 1979). But food storage also requires the animal not only to remember that they stored food but also to relocate the food when it is needed. For larder hoarders, such as some rodents, store or cache relocation may simply mean returning to their burrow or a nearby larder. Straightforward relocation, but probably requiring investment in defence of such a valuable commodity. Scatterhoarders avoid the need for cache defence by doing what it says on the tin: they disperse pieces of excess food around the environment so that if another individual happened to stumble across a piece hidden by the scatterhoarder, it would be no better informed as to the location of the next nearest cache (Stapanian and Smith 1984). But the problem of defence is replaced by a problem that seems no less challenging: that of coming up with an algorithm for concealment that is not obvious, or even more challenging, remembering each of the cache locations. Challenging because the avian food storers store anything from hundreds to thousands of food items, and some species do not retrieve their caches for several months. The pay-off in this latter case is that the storer with a good algorithm or good memory has access to food not just in times of scarcity such as the winter months but can also get themselves into breeding condition early (Brodin 2010).

Although it seemed implausible that scatterhoarders might use memory to relocate their caches due to the number of items they stored, field data showed that it was at least possible (Cowie et al. 1981). But showing that it was possible does not mean it was easy to demonstrate definitively (and still is not). Very soon, because some of these species would also store and retrieve in a laboratory setting, experimental manipulations in the laboratory became the locus of testing (e.g. Kamil and Balda 1985; Olson et al. 1995; Sherry et al. 1981; Shettleworth and Krebs 1982, 1986; Shettleworth et al. 1988). That these birds did use memory of locations to store their food in preference to using search strategies and that they used visual cues to do so soon became pretty clear (Sherry 1984). But the discovery that damage to the hippocampus of food-storing chickadees specifically affected their ability to retrieve their stored food (Sherry and Vaccarino 1989), together with evidence that the size of the hippocampus varied across species in relation to food storing (Krebs et al. 1989; Sherry et al. 1989), gave a new edge to the investigation of the nature of the memory involved in relocation of stored food: interspecific comparations of cognitive abilities, especially in the context of spatial learning and memory became the new focus. Whether or not food-storing birds were smarter than were non-storers was not the question, but both the added demand for spatial memory and the evidence that a key part of the brain was larger strongly suggested that food storers should have better spatial memory than should non-storers. In the context of Macphail’s (1982) critique of comparative cognition, the specific cognitive ability and the access to comparisons of species expected to have a greater demand for that ability and closely related species that do not face this demand seemed promising. The phylogenetic proximity might mean that some of the extrinsic factors (varying from perceptual abilities, through attention and motivation) discussed by Macphail were reduced, or even absent.

And it is from this point that the history, as well as the ultimate endpoints, of the experiments comparing the cognitive abilities of food storers with closely related non-storers hold value for researchers working on comparative cognition, especially those interested in adaptation and cognition. No single experiment really stands out as pivotal, but the series of discoveries (and sometimes lack thereof) was, and still is, a lesson in the road to finding evidence to support a clear, compelling, even appealing, hypothesis: it is not always that easy. And in comparative cognition, it may be especially challenging. Macphail was not wrong: there are always plausible alternative explanations, even if the experimental outcome supports the original hypothesis. But along the way, a lot may be learned about cognitive abilities of the comparison species, as well as by the researchers in designing good or useful experiments.

One cognitive feature was shown via multiple experiments in different laboratories, was that food storers performed well on spatial memory tasks other than food storing (Herz et al. 1994; Hilton and Krebs 1990; Hitchcock and Sherry 1990; Krebs et al. 1990; Shettleworth et al. 1990), so the ability to remember food stores was a transferable skill. What was less clear was how much better were the spatial memory capacities of food storers than those of non-storers: repeated experiments comparing storers ability to relocate rewarded locations with non-storers failed to come up with consistently compelling evidence that the two groups of birds always differed in their spatial memory abilities (e.g. Healy 1995; Healy and Krebs 1992a, b).

These comparisons were somewhat hampered by the fact that for several research groups the non-storing species were not especially closely related to the storing species. North American chickadees and corvids might vary interspecifically in the amount of food they store, and the duration before which the food is retrieved, but all of them store food to some degree. And while there is variation in hippocampal volume depending on the degree of storing (both amount of food and duration of storage: Basil et al. 1996; Gould et al. 2013; Hampton et al. 1995; Healy and Krebs 1992c), the biggest difference in hippocampal volume is that between storers and non-storers. One might expect that this should, then, lead to a bigger difference in cognitive performance, especially in spatial memory, between storers and non-storers. But no matter what kind of spatial memory test, be it more ‘natural’ whereby birds flew around rooms searching, and then relocating, food (e.g. Healy and Krebs 1992a; Hilton and Krebs 1990; Krebs et al. 1990), or were tested with more traditional kinds of tests such as delayed matching to sample (e.g. Healy and Krebs 1992b) or tests of proactive interference (e.g. Hampton et al. 1998), the differences in performance did not seem to be of the expected magnitude. While there was often a tendency for food storers to out-perform non-storers, this apparent difference did not often reach significance.

The most obvious differences, instead, came from preferences for cue use, whereby food storers seemed to rely much more heavily on so-called spatial cues (e.g. the location of a rewarded feeder) relative to visual cues (the colour of a rewarded feeder) than did non-storers when the two cue types were put in conflict during food retrieval sessions (Brodbeck 1994; Brodbeck and Shettleworth 1995; Clayton and Krebs 1994b). Sometime later, however, even this preference for spatial over visual cues was shown not to be specific to food-storing species: non-storing great tits *Parus major* also prefer spatial over visual cues once they have had multiples experiences of a rewarded location (Hodgson and Healy 2005) and food-storing mountain chickadees *Poecile gambeli* prefer colour over spatial cues when relocating food in an associative learning task (LaDage et al. 2009).

While the behavioural evidence for differences in spatial memory between storers and non-storers was equivocal, confirmation that the hippocampus really was key to the relationship between spatial memory and food storing was provided by evidence that the size of the hippocampus of food-storing birds grew in relation to food-storing experience (Clayton and Krebs 1994a; Healy et al. 1994; Healy and Krebs 1993). While the neural bases of this growth (e.g. increased cell spacing, cell size, more cells, or some other) are not yet well understood, data provided continuing impetus to the search for ‘cognitive corroboration’ that hippocampal volume was a good representation of cognitive capacity. But it did take quite a bit more work before such data appeared, and when they did, they showed that a larger hippocampus did not necessarily confer better spatial memory across the board. Rather it appeared that only some components of spatial memory were better and, in the tits at least, these were specific to duration of spatial memory, rather than to capacity: food-storing tits could remember even a single location for longer than could non-storing tits (Biegler et al. 2001; McGregor and Healy 1999). It should be noted here, however, that the durations tested in these experiments, as for many of the laboratory experiments, were not ‘real-world’ with respect to the durations over which food-storing birds typically leave stores before retrieval.

Around the time these data were collected, the difficulty for North American researchers of finding closely related non-storing species for comparison with storing species (note that there was quite a lot of work on corvids that differed in their dependence on food-storing, e.g. Balda et al. 1997; Bednekoff et al. 1997) was reduced by the discovery that different populations of a food-storing species *Poecile atricapilla* differed in their performance on spatial tasks: black-capped chickadees from Alaska stored more food and recovered a greater proportion of their caches than did black-capped chickadees from Colorado. Furthermore, the Alaskan chickadees had a larger hippocampus than did the Colorado chickadees (Pravosudov and Clayton 2002). The relationship between climatic severity and hippocampal volume, with a presumed variation in dependence on stored food, and memory capacity, was later also seen along a gradient of environmental harshness from Alaska to Kansas, in wild and captive-raised birds (Roth et al. 2012; Roth and Pravosudov 2009), and then in an altitudinal gradient in mountain chickadees *P. gambeli* (Freas et al. 2013, 2012). These data from laboratory and wild contexts confirming each other have formed the basis for a continuing set of insights regarding food storing, spatial memory, and hippocampal structure. For example, there is now evidence for place cells in the hippocampus on food storers (Payne et al. 2021). In addition, the genetic basis for variation among the populations is being described (e.g. Branch et al. 2022; Pravosudov et al. 2012, 2013), experimental tests of spatial memory of food storers in the wild are finally occurring (e.g. Croston et al. 2016; Shaw et al. 2015; Sonnenberg et al. 2019; Tello-Ramos et al. 2018), as well as evidence for survival (Branch et al. 2019b) and selective advantages to better spatial memory (Branch et al. 2019a; Shaw et al. 2019). It may have taken a while but here is the confirmation of the pieces needed to show that food storing selects for better spatial memory and a bigger hippocampus (Krebs 1990; Smulders et al. 2010). This body of work also provides precision to the loose assumption that a big brain means a smarter animal. In this case, not a big brain, ‘just’ a bigger hippocampus, and not smarter, ‘just’ with better spatial memory. Natural selection can, and does, act rather precisely on specific parts of the brain and researchers particularly interested in evolution and animal cognition might consider how these data help them in thinking about the cognitive questions they ask of their own animals. Specifically, whether adding a neural component to their work might not also be beneficial.

## Episodic memory

Who knows where such an investigation might lead? After all, work on food storing, spatial memory and the hippocampus also led to one of the most impactful discoveries in animal cognition, a real step change, which was the discovery that animals had episodic-like memories (Clayton and Dickinson 1998). This work enabled animal cognition researchers to first imagine and then to design useful experiments to look for cognitive capabilities in their animals that had previously not apparently been within scope (e.g. cuttlefish Jozet-Alves et al. 2013). There have since been enthusiastic searches for episodic-like memory, which have resulted in serious consideration of experimental designs and of types of memory (e.g. Babb and Crystal 2005; Bonardi et al. 2021; Eacott and Norman 2004; Feeney et al. 2009; Zhou and Crystal 2011). Some of these involve what-where-when memories, others what-where-which memories, leading to the discovery that a wide range of animals, in the wild as well as in the laboratory, can integrate all or some of the components of episodic-like memory (e.g. Janmaat et al. 2013; Jelbert et al. 2014; Lo and Roberts 2019; Marshall et al. 2013; Roberts et al. 2008; Zinkivskay et al. 2009). These investigations will be much aided by technological advances both in the laboratory (automated tracking of storing and retrieval: Applegate and Aronov 2022) and in the field (neurologgers: Ide and Takahashi 2022).

If an animal has memories of its past such as what-where-when memories, this raises the question as to whether the animal might use them to plan its future. After all, what value is a memory if not to aid in a decision right now, or to make a decision now that might help the animal to deal with an upcoming situation. Once the question was asked, again food storers provided the initial (Raby et al. 2007) and later (Gould et al. 2012) data that have led to a wealth of debate and new experiments directed at the cognitive capacities of animals. Perhaps disappointingly for discussions that began in the context of natural selection, some of this debate has resulted in hierarchical interpretations of cognitive capabilities (e.g. feathered apes, Emery 2004). But the more important point is that the field of animal cognition is a long way from its origins and the work on food storing, spatial memory, and hippocampus (and the song learning literature) helped the field get to episodic-like memory and on to planning (the process of deciding in detail how to do something before one actually starts to do it). But this is also a point at which the neural analyses have tended not to have accompanied the behavioural data. Some pertinent neural analyses are proceeding but are currently dissociated from animal cognition and addressed to humans (e.g. Inostroza et al. 2013; McCormick et al. 2018).

## Nest building

The food storing, spatial memory, hippocampus literature has directly led to methodological and empirical advances in animal cognition (as above). It is also now indirectly shaping investigation into a rather different behaviour, nest building in birds. Although this is a behaviour that for many remains stubbornly associated with innateness (e.g. Anholt 2020), over the past decade in particular, evidence has accumulated steadily for a role for learning and memory in varying aspects of nest building. Much of the work has been focussed on a ‘model’ species in the laboratory, as the food-storing investigation began by being directed at just food storers and in the laboratory, because of the logistic problems of investigating the behaviour experimentally in the field. In the case of nest building, the zebra finch *Taeniopygia guttata* is the model, not least because it builds very readily under laboratory conditions, and with a wide variety of materials (from strips of coloured coconut fibre or paper, through pieces of coloured string or wool, to materials held together by wire). It is also sometimes useful that having built one nest, a male zebra finch (the male is the primary nest builder in this species) will, if the finished nest is removed, almost immediately begin and proceed to complete another nest. Furthermore, zebra finch offspring become reproductively active at around three months old. Various attributes of building/building performance can then be examined/quantified, including choice/preferences of material, amount of building, the success of building choices, and, of course the feature of nest building that has received some attention in the past, the morphology of the nest (typically weight but also dimensions such as height, depth, and breadth).

As it was for food storing, the first experiments with nest building were addressed at determining what role learning and memory might play. Then and since, all of the experimental work on nest building in zebra finches has contributed confirmation that the birds learn and remember a variety of features of material or the outcome of reproductive events. For example, males that build a nest with material of a colour they do not prefer will, if they successfully raise offspring from that nest, choose material of that colour to build their second nest (Muth and Healy 2011).

Sticking with materials that work was also seen in a test of whether birds will build to the ambient temperature. This has been proposed to explain why birds building in more northerly, higher, or cooler environments often build bigger nests and/or with thicker walls than do conspecifics building in more temperate climes (e.g. Crossman et al. 2011; Deeming et al. 2012; Mainwaring et al. 2014). And indeed, under experimental conditions zebra finches will build a nest that is bigger when the ambient temperature is cooler (contains more pieces of string/material: Campbell et al. 2018; Edwards et al. 2020b). But in the Edwards et al. (2020a, b) experiment, some of the birds were switched to a room with the other temperature to build a second nest: half of those that had built their first nest at 18 °C built their second nest at 30 °C, and vice versa, and half the birds built their second nest at the same temperature as that at which they had built their first. At first the data appeared to confirm that comment that one should never repeat a successful experiment because the birds did not build their second nest in response to the temperature. But for an animal cognition researcher, the birds did something said researcher might appreciate, because the second nest built by the birds depended on how well they had done with their first nest with regard to reproduction. Those birds that had produced offspring with their first nest, put the same number of pieces of material into their second nest as for their first. The birds that had not been reproductively successful with their first nest all put more material into their nest. In both parts of the experiment, adding more pieces of material to the nest increased the nest temperature, and increased the probability of reproductive success. Just as in the earlier experiment in which builders had associated the colour of materials with the success of their nest, in this latter experiment birds associated either the number of pieces of material, or the temperature the nest reached with nest success.

There have been several other experiments that show birds choose among materials for their nest, for example, choosing material of the colour that matches the nest box and the walls of their cage (the first experimental evidence that birds camouflage their nests: Bailey et al. 2015) or choosing more rigid over flexible material. This choice of rigid material is sensible as it can take around half as many pieces of rigid material to build a nest than it takes with pieces of flexible material (Bailey et al. 2014). Zebra finches building in a nest box with a relatively small entrance hole will initially choose pieces of material that they can easily take into the box. However, with experience they will alter how they handle the material so that they can use all of the materials provided for building their nest (Muth and Healy 2014). Data from the wild also show that as weaver birds build more they improve their handling skills, and drop fewer pieces of grass (Walsh et al. 2013).

Increasing evidence, then, that nest building involves a lot more learning and memory than is usually assumed for this widespread avian behaviour. Like food storing, there is the possibility for taking a comparative approach because some birds do not build a nest at all (as non-storing birds do not store food; e.g. Emperor and King penguins *Aptenodytes forsteri* and *A. patagonicus*, common murre *Uria aaige*, and obligate brood parasites such as cuckoos), while some build apparently simple nests, some build nests that seem apparently more complex (e.g. domed nests as built by zebra finches, woven nests as built by weavers, nests stitched together by tailor birds or felted by penduline tits). Still others build together (e.g. sparrow weavers), or build alone but add their nest to the local ‘apartment block’ (the sociable weavers). This variety offers rich potential for examining variation on physical cognition and social cognition, as well as learning and memory more generally.

## Neuro + nest building

Investigating the neural basis of food storing was helped by the structural and functional work on the mammalian hippocampus that had both preceded and was concomitant with the avian hippocampus work. In particular, there was clear support for the role of the hippocampus in spatial learning and memory, and for homology between the mammalian and avian hippocampus. For nest building, there is considerably more work to be done with neural correlates (Hall et al. 2015). But there are now data that implicate at least two pertinent brain regions. Some of these data are comparative, on cerebellum size and surface folding (foliation). The function of the vertebrate cerebellum (data mostly from mammals) is to control motor output especially manipulative abilities and may also play a role in cognition (Coolidge 2021; Habas 2022; Honda et al. 2018). These roles are consistent with the finding that as nest complexity (as quantified into three very simplified categories: no nest, platform nest, cup nest) increases so does cerebellar foliation (Hall et al. 2013).

Immediate early gene activation (used as a molecular marker of neuronal activity) data have now implicated several brain regions in nest building. For example, there is greater activity in the anterior motor pathway, which underpins sequential motor actions, and in the dopaminergic reward system as builders pick up and take more pieces of material to their nest (Hall et al. 2014). There are also increases in activity in the social network and some cerebellar folia with various building behaviours such as carrying material, depositing material, and tucking material into the nest structure (Edwards et al. 2020a). Thus far all of these neural data show roles for motor output and reward rather than clearly contributing to understanding the cognitive components of nest building.

Being in a position to predict where to look in the brain for a cognitive signal of nest building will require both better functional understanding of relevant avian brain regions and a clearer description of the particular cognitive abilities required to build a nest. Both of these are far from straightforward. The first will require major efforts by avian neuroscientists, who will need to be encouraged to find the necessary functional work interesting. Neuroendocrinologists might, at least, find the fact that in many species the builder is one sex or the other of some interest. For example, in zebra finches the male is the builder while in tits the female does the building. Because in zebra finches the females are sometimes observed to do some or nearly all the building, there seems a very plausible argument that sex hormones are involved in some way. But while there are some hormonal data on a range of behaviours considered to be ‘nesting’ behaviours, there are as yet, few data on a causal role for the sex hormones in nest building.

For animal cognition researchers the job will be to develop a more precise description of the cognitive abilities required for nest building. Physical cognition might loosely appear to describe nest building (for those who are not convinced the behaviour is largely or entirely innate), but physical cognition is still a rather diffuse description, and one that has not yet led itself to clear predictions about its neural bases. The focus for physical cognition researchers is currently heavily on tool use, and behavioural descriptions of the species, individuals, and contexts in which it occurs are increasing in appearances in the literature. There are some pertinent neural data on tool use in primates (e.g. McDowell et al. 2018; Tia et al. 2017) but these use technologies that are not yet appropriate for examining nest building. But given that builders will observe others, and change their building decisions as a result (if the birds they observe are familiar: Guillette et al. 2016), and perhaps even more usefully, will respond to videos of building (Guillette and Healy 2019) and even views of a nest (Breen et al. 2019; Camacho-Alpizar et al. 2021), perhaps functional scanning of nest builder brains may not be too far in the future. Furthermore, although zebra finches do not appear to imprint on to the colour of the material of their natal nest (Muth and Healy 2012; Sargent 1965), they do learn about the colour of material to which they are exposed during post-fledging juvenile development (Breen et al. 2020), which opens a window of opportunity for examining neural function during this developmental stage.

## Concluding remarks

The food storing, spatial memory, hippocampus work led to significant contributions in our understanding of all three components, behaviour, cognition, and neurobiology. These contributions did not come without challenges especially in adding the neurobiology to the cognition. But given that the value of putting the neuro together with the behaviour and cognition is so clear in the food-storing case, I hope that it will continue to inspire researchers in animal cognition to consider attempts to do likewise for their own favoured system.

## Data Availability

There are no original data contained within this paper.
